# The value of pharmacist medication therapy management of capecitabine

**DOI:** 10.3389/fphar.2025.1600976

**Published:** 2025-10-15

**Authors:** Jing Xue, Chengcui Huang, Xiaorui Liu, Xiaoyu Xie, Xiaoyan Li

**Affiliations:** ^1^ Department of Pharmacy, The Sixth Affiliated Hospital, Sun Yat-Sen University, Guangzhou, China; ^2^ Biomedical Innovation Center, The Sixth Affiliated Hospital, Sun Yat-Sen University, Guangzhou, China; ^3^ Department of Pharmacy, Affiliated Cancer Hospital and Institute of Guangzhou Medical University, Guangzhou, China; ^4^ Department of Medical Oncology, The Sixth Affiliated Hospital, Sun Yat-Sen University, Guangzhou, China

**Keywords:** capecitabine, pharmacist, medication therapy management, combined oncologist-pharmacist clinic, outpatient

## Abstract

**Objectives:**

This study aims to evaluate the value of the medication therapy management (MTM) provided by an oncologist - pharmacist joint clinic for patients self - administering capecitabine, and to identify intervention programs tailored for pharmacists.

**Methods:**

A total of 200 patients were included in the study and were followed up for 1 year. Among them, 100 received MTM and the other 100 were considered a control group. A retrospective, longitudinal comparison of adverse effects (AEs) in capecitabine patients who receive MTM vs. those who do not. During this period, pharmacists systematically reviewed aspects such as drug indications, usage and dosage, as well as the improvement and resolution of AEs, and report the identified problems to the doctor, and discuss with the doctor about prescribing medications for the patient for prevention or treatment. With a particular focus on evaluating the improvement of patients’ AEs during the follow - up stage.

**Results:**

Our research results indicate that the gastrointestinal tract (χ^2^ = 26.868, p = 0.000) is a common site for AEs, with particularly notable differences observed in symptoms such as anorexia and nausea. Furthermore, significant differences in AEs affecting the central and peripheral nervous systems (χ^2^ = 20.864, p = 0.000) are evident between the two groups, especially concerning insomnia symptoms. Among the hematological AEs, the most pronounced phenomenon is the decrease in hemoglobin levels (χ^2^ = 21.333, p = 0.000). Moreover, pharmacist intervention can lower the incidence of pain and leukopenia. There was no significant difference in the levels of the various tumor markers between the two groups.

**Conclusion:**

In the joint outpatient service model of oncologists and pharmacists, pharmacists can manage patients taking oral capecitabine through MTM. This measure can reduce the incidence of drug AEs related to capecitabine, especially those in the gastrointestinal tract, nervous system and blood system, thereby enhancing the safety of patients’ medication.

## Introduction

The postoperative adjuvant chemotherapy regimen for cancer patients has evolved. It has shifted from relying solely on intravenous treatment to a combination of intravenous and oral chemotherapy. The National Comprehensive Cancer Network (NCCN) reported that capecitabine, which was approved for marketing in 1998, ushered in a new era of oral chemotherapy drugs (Weingart et al., 2008). There has been a remarkable increase in the utilization of oral chemotherapy agents (Chen et al., 2020). Patients tend to prefer oral chemotherapy due to its convenience. It minimizes the disruption of treatment to their work and social activities and obviates the need for frequent hospital visits (Increased Use of Oral Chemotherapy, 2008; Liu et al., 1997; Simchowitz et al., 2010). During intravenous chemotherapy, medical staffs are in charge of overseeing drug efficacy, AEs, and treatment adherence. In contrast, when patients administer oral chemotherapy drugs at home, they transition from being under strict medical supervision to self - managing their treatment. This heavy reliance on patients for self - management can potentially result in issues such as low treatment adherence, medication errors, and the occurrence of AEs and interactions (Hershman et al., 2011; Solomon et al., 2019; Jacobs et al., 2019).

A clinic where an oncologist collaborates with a pharmacist is a unique environment in which the oncologist performs patient consultations alongside a pharmacist who provides MTM (Pellegrino et al., 2009; Association and Foundation, 2008). This collaborative approach between oncologists and pharmacists has the potential to serve as a new healthcare delivery model that addresses issues concerning the safety, efficacy, and adherence to capecitabine. MTM is a new type of pharmaceutical service model, which originated in the United States. It refers to a series of specialized services provided by pharmacists with superior pharmaceutical expertise to patients, including medication education, AEs management and consultation guidance, in order to enhance patients’ medication compliance, prevent medication errors, reduce the incidence of AEs and ultimately achieve the goal of self-management by patients and improvement of treatment outcomes (Wondesen et al., 2022). MTM has been successfully utilized in the treatment of various chronic conditions, and the partnership between healthcare providers such as physicians and pharmacists indicates an emerging service model that is progressively gaining popularity (Liu et al., 1997; Liu et al., 2021a; Lu et al., 2023; Ulrich et al., 2019; Hadi et al., 2012; Hirsch et al., 2017) However, the specific benefits of MTM for patients undergoing capecitabine remain to be conclusively established (de Oliveira et al., 2020).

The objective of this research was to compare the frequencey of adverse effects in patient that underwent MTM vs. those that did not. This will reflect whether the drug treatment management provided by the outpatient clinic established through collaboration between oncologists and pharmacists is necessary for patients taking capecitabine, and determine the possible intervention measures that pharmacists may need to implement.

## Materials and methods

### Study design and setting

We conducted a retrospective study to assess whether the incidence of chemotherapy-related AEs in patients who received capecitabine treatment and received MTM services provided by pharmacists was lower than that in patients who did not receive such services. The study was conducted at the Sixth Affiliated Hospital of Sun Yat-Sen University between 10 May 2023 and 10 June 2023.

### Sample size calculation

The sample size was calculated based on the primary outcome of AEs management. The research indicates that in the MTM studies led by pharmacists, the intervention rate for AEs was 35.8% (Zhang et al., 2021a). A study on an oral chemotherapy management program indicates that the management of capecitabine has reduced the incidence of any grade AEs from 70.3% to 58.9% (a decrease of 11.4%) (Nhean et al., 2021). A study on the implementation of MTM for community patients with chronic diseases indicates that the incidence of AEs has decreased from 32.0% to 9.3% (a reduction of 22.7%) (Yuan ling et al., 2020). Therefore, in this study, assuming a baseline ADE rate of 30% in the standard care group and an expected reduction to 15% in the combined clinic group was required. Based on the average number of patients visiting the combined oncology and medicine clinic each month, which is approximately 130, considering factors such as incomplete patient data, non-compliance with inclusion or exclusion criteria, we have set the number of patients in the intervention group using capecitabine to be 100. According to the number of patients planned to be included in the intervention group, we set the control group to include an equal number of patients. The intervention group was a convenience sample of clinic patients. Therefore, a total of 200 patients were included in the study. Patients who visited the oncology and pharmaceutical joint clinic from May 10th to 10 June 2023 and were using capecitabine were selected as the intervention group. Patients who were using capecitabine and were solely treated by the same doctor during the same period were chosen as the control group. Patients were first included in the study according to the chronological order of their visits, and then further confirmed in line with the study’s predefined inclusion and exclusion criteria. The studies involving human participants were reviewed and approved by the Institutional Review Board of the Sixth Affiliated Hospital of Sun Yat-sen University (approval no. 2022ZSLYEC-622). The study was conducted according to the guidelines of the Declaration of Helsinki. This study was retrospective; thus, an application was made to exempt patients from signing informed consent.

### Inclusion and exclusion criteria

Patients aged 18 years or older, diagnosed with cancer, and receiving capecitabine were included. Patients with incomplete follow-up records or those receiving non-capecitabine were excluded.

### Combined oncologist-pharmacist clinic model

**FIGURE 1:**
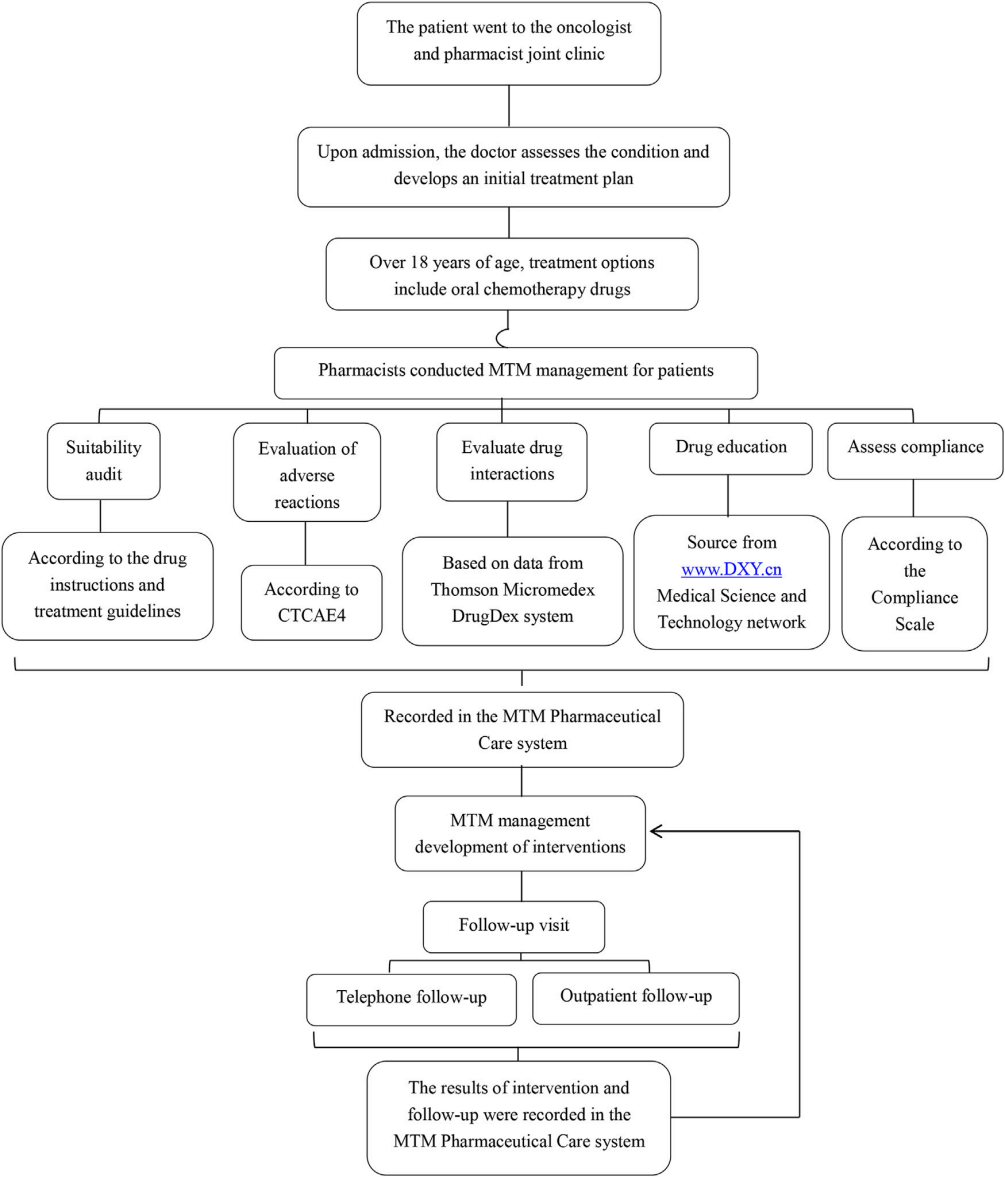
Working mode of oncologist and pharmacist joint clinic.

The combined clinic model involved both oncologists and pharmacists providing comprehensive care. The oncologist and the pharmacist were in the same consultation room. The pharmacist did not haveindependent prescribing authority. Patients first visited the oncologist for medical advice and were then referred to the pharmacist for MTM. Figure 1 shows the working mode of the oncologist and pharmacist joint clinic. The pharmacist’s intervention measures included:

1. Reviewing indications, use, and dosage of capecitabine and adjuvant therapeutic drugs.Once it was determined through assessment that the patient’s medication dosage might need to be adjusted, the pharmacist provided on-site suggestions. The doctor then reviewed the patient’s information again to decide whether to make the modification. For patients whose information was reviewed again and it was found that modification was necessary, the doctor reissued the prescription.
2. Evaluate AEs, interactions and adherence. For AEs that truly require drug prevention or treatment, pharmacists will provide on-site advice, and then doctors will decide whether to prescribe the corresponding drugs.
3. Monitoring laboratory data and managing symptoms or disease progression.
4. Providing written and verbal medication education.

### Follow-up procedures

Patients were followed up for 1 year. Both groups of patients obtained their test results (such as blood routine, liver function, etc.) through the electronic medical record system. The intervention group patients received on-site follow-up by pharmacists. For patients who failed to keep the appointment on time and were unable to undergo on-site follow-up, the pharmacist would conduct follow-up via phone and send a standardized electronic form to the patients for them to fill out, in order to collect information on the occurrence and severity of clinical medical symptoms such as diarrhea, nausea, and insomnia. The control group patients received personalized routine treatment from the same oncologist. The patients in the control group filled out the standardized paper form on-site to collect information on the occurrence and severity of clinical medical symptoms. The content of the standardized electronic form was consistent with that of the standardized paper form. The standardized electronic/paper forms were designed according to CTCAE 4.0, and the severity of adverse events was determined based on the CTCAE 4.0 standard. The follow-up included:

1. Clinical medical symptoms, such as nausea, vomiting, diarrhea, insomnia, etc.
2. Laboratory data, such as blood routine, liver function, etc.

### Outcome indicators

The main evaluation indicators are the frequency and severity of adverse events.

### Data collection and analysis

The data were collected through the electronic medical record verification system, on-site standardized paper-based forms for follow-up, and telephone standardized electronic forms for follow-up. For qualitative data such as clinical medical symptoms, descriptive stats were first used to show distribution (frequencies, percentages), followed by chi - square tests to find associations. For continuous variables, t - tests were applied if data met normality and variance assumptions; otherwise, non - parametric tests were used. All analyses were performed using SPSS software (version 26.0).

## Results

A total of 200 patients were included in the study, all of whom were followed up for 1 year. The baseline characteristics of the two groups were comparable, with no significant differences observed in gender, age, tumor type, capecitabine dosage, or treatment regimens. For details, please refer to Table 1.

**TABLE 1:** Baseline characteristics of patients.

Item	Intervention group (n = 100)	Control group (n = 100)	χ^2^/t	p
Gender (Male)	63 (63.0%)	55 (55.0%)	1.323	0.250
Age	55.21 ± 11.70	57.60 ± 12.24	1.412	0.160
Primary tumor type			0.255	0.614
Colorectal cancer	97 (97.0%)	99 (99.0%)		
Gastric cancer	3 (3.0%)	1 (1.0%)		
Capecitabine dose	2.94 ± 0.40	2.98 ± 0.59	0.562	0.575
Combined use with other anti-cancer medications^a^	22 (22.0%)	21 (21.0%)	0.030	0.863
Combined use with other chemotherapy medications^b^	51 (51.0%)	60 (60.0%)	1.640	0.200

^a^ The “other anti-cancer medications” referred to here are bevacizumab, fraxinib, or regorafenib.

^b^ The “other chemotherapy medications” referred to here are oxaliplatin or irinotecan.

On average, the number of visits for patients in the intervention group and the control group was the actual average value and standard deviation, and the range was between 1 and 3 times. In the intervention group, 60% of the patients received the intervention measures at the combined outpatient clinic. The intervention group reported a total of 685 cases of ADE, while the control group reported 979 cases. The incidence of AEs decreased by 30.0%. Table 2 shows comparative data on AEs between the intervention and control groups. In the gastrointestinal system, the intervention group reported 252 cases while the control group reported 361, indicating a significant difference (χ^2^ = 26.868, p = 0.000). Notable AEs such as anorexia, nausea, vomiting, diarrhea, and oral mucositis/ulcers exhibited significant differences between the groups. For skin-related AEs, the intervention group had 68 cases compared to 81 in the control group, with no significant difference observed (χ^2^ = 1.394, p = 0.238), except for alopecia which showed a significant difference (χ^2^ = 5.107, p = 0.024). In the central and peripheral nervous systems, the intervention group had 167 cases while the control group had 242, revealing a significant difference (χ^2^ = 20.864, p = 0.000). Significant differences were noted for insomnia, dizziness, hand-foot syndrome, and headache, while fatigue and drowsiness did not show significant differences. Regarding systemic AEs, the intervention group reported 55 cases and the control group 71, with no significant difference found (χ^2^ = 2.572, p = 0.109), including symptoms such as fever, fatigue, and pain. There were 16 cases in the intervention group and 18 in the control group suffering conjunctivitis/increased tearing, with no significant difference (χ^2^ = 0.142, p = 0.707). In the hepatic and biliary system, the intervention group had 43 cases while the control group had 58, with no significant difference (χ^2^ = 2.550, p = 0.110), except for alkaline phosphatase elevation which demonstrated a significant difference (χ^2^ = 5.329, p = 0.021). For endocrine system impairment, both groups reported 18 cases with no significant difference (χ^2^ = 0.000, p = 1.000), similar findings were observed for hypercalcemia, hypocalcemia, and hypokalemia. In the hematological system, the intervention group had 66 cases compared to 130 in the control group, indicating a significant difference (χ^2^ = 24.978, p = 0.000). Significant differences were noted for hemoglobin decrease and lymphocytopenia, while other indicators did not show significant differences. Others like alopecia (p = 0.024) and ALP elevation (p = 0.021) were also had significant differences between two groups.

**TABLE 2:** Adverse effects (AEs) performance.

Category	Item	Intervention group	Control group	χ^2^	*p*
Gastrointestinal system		252	361	26.868	0.000
		22.91 ± 10.23	35.82 ± 24.17	1.252	0.225
	Anorexia	41	73	20.889	0.000
	Nausea	33	59	13.607	0.000
	Vomiting	5	14	4.711	0.030
	Abdominal pain	17	12	1.008	0.315
	Diarrhea	23	59	26.788	0.000
	Abdominal bloating	24	14	3.249	0.071
	Dry mouth	30	42	3.125	0.077
	Oral mucositis/ulcers	31	51	8.268	0.004
	Dysgeusia	17	12	1.008	0.315
	Constipation	16	17	0.036	0.849
	Indigestion	15	8	2.407	0.121
Central and peripheral nervous system		167	242	20.864	0.000
		27.83 ± 14.25	40.33 ± 12.64	1.607	0.139
	Fatigued	49	54	0.500	0.479
	Drowsy	30	24	0.913	0.339
	Insomnia	23	49	14.670	0.000
	Giddy	20	35	5.643	0.018
	Hand-Foot syndrome	37	51	3.977	0.046
	Headache	8	29	14.624	0.000
Hematological system damage		66	130	24.978	0.000
		11.00 ± 9.86	21.67 ± 18.47	1.248	0.240
	Prolonged activation of partial thromboplastin	2	6	2.083	0.149
	Leukopenia	20	26	1.016	0.313
	Decreased hemoglobin	24	56	21.333	0.000
	Lymphocytopenia	0	21	23.464	0.000
	Neutrophil count decreased	14	8	1.839	0.175
	Thrombopenia	6	13	2.850	0.091
Skin AEs		68	81	1.394	0.238
		17.00 ± 9.56	20.25 ± 3.10	0.647	0.542
	Pruritus	21	23	0.117	0.733
	Rash	13	22	2.805	0.094
	Dry skin	28	20	1.754	0.185
	Alopecia	6	16	5.107	0.024
Systemic AEs		55	71	2.572	0.109
		18.33 ± 16.26	23.67 ± 20.03	0.358	0.738
	Fever	4	3	0.148	0.700
	Fatigue	36	43	1.025	0.311
	Pain	15	25	3.125	0.077
The hepatic and biliary system		43	58	2.550	0.110
		18.33 ± 16.26	23.67 ± 20.03	0.800	0.454
	ALT elevation	15	20	0.866	0.352
	AST elevation	16	18	0.142	0.707
	ALP elevation	0	7	5.329	0.021
Endocrine system impairment		18	18	0.000	1.000
		6.00 ± 5.20	6.00 ± 4.36	0.000	1.000
	High calcium	0	3	1.354	0.245
	Low calcium	9	4	2.057	0.152
	Low potassium	9	11	0.222	0.637
AEs of the ear, nose, throat and facial features and organs		16	18	0.142	0.707
	Conjunctivitis/Increased tearing	16	18	0.142	0.707

The numbers in Table 2 represent the number of AEs, for each research group.

Moreover, based on the CTCAE 4.0 scale, significant statistical differences are observed in the aspects of pain (χ^2^ = 4.571, p = 0.033) and leukopenia (χ^2^ = 3.982, p = 0.046) between the intervention group and the control group (Table 3).

**TABLE 3:** AEs performance according to CTCAE.

Item	Intervention group	Control group	χ^2^	p
Abdominal bloating			0.000	1.000
Ⅰ-Ⅱ	20 (83.3)	12 (85.7)		
Ⅲ-Ⅳ	4 (16.7)	2 (14.3)		
Dry mouth			0.030	0.862
Ⅰ-Ⅱ	29 (96.7)	39 (92.9)		
Ⅲ-Ⅳ	1 (3.3)	3 (7.1)		
Pruritus			0.030	0.862
Ⅰ-Ⅱ	16 (76.2)	17 (73.9)		
Ⅲ-Ⅳ	5 (23.8)	6 (26.1)		
Dry skin			0.159	0.690
Ⅰ-Ⅱ	26 (92.9)	17 (85.0)		
Ⅲ-Ⅳ	2 (7.1)	3 (15.0)		
Fatigued			0.190	0.663
Ⅰ-Ⅱ	44 (89.8)	47 (87.0)		
Ⅲ-Ⅳ	5 (10.2)	7 (13.0)		
Drowsy			0.135	0.713
Ⅰ-Ⅱ	26 (86.7)	19 (79.2)		
Ⅲ-Ⅳ	4 (13.3)	5 (20.8)		
Fatigue			0.000	1.000
Ⅰ-Ⅱ	32 (88.9)	38 (88.4)		
Ⅲ-Ⅳ	4 (11.1)	5 (11.6)		
Pain			4.571	0.033
Ⅰ-Ⅱ	13 (93.3)	14 (56.0)		
Ⅲ-Ⅳ	1 (6.7)	11 (44.0)		
Leukopenia			3.982	0.046
Ⅰ-Ⅱ	17 (85.0)	15 (57.7)		
Ⅲ-Ⅳ	3 (15.0)	11 (42.3)		

In table 3, I, II, III, and IV, represent the classification levels of AEs. This classification standard corresponds to the classification standard in CTCAE, 4.0. Levels I-II, indicate mild to moderate AEs, while levels III-IV, represent severe AEs.

During the follow-up period, we also monitored the status of relevant tumor markers, including CEA, CA12-5, CA19-9, CA15-3, and AFP. After 6 months of follow-up, there was no significant difference in the levels of the various tumor markers between the two groups.

## Discussion

### The transformation of the pharmacist’s pharmaceutical care model

In China, with the evolution of national-level pharmaceutical service standards, the model of pharmaceutical services has undergone significant changes. In 2017, the “Notice on Strengthening Pharmaceutical Management and Transforming the Model of Pharmaceutical Services” proposed to shift the focus of pharmaceutical services from “centering on drugs” to “centering on patients”, and from “centering on ensuring drug supply” to “ensuring drug supply while focusing on strengthening pharmaceutical professional technical services and participating in clinical medication” (O.o.t.N.H.a.F.P.C.O.o.t.N.A.o.T.C. Medicine, 2017). Our research is based on the joint outpatient clinics of oncologists and pharmacists (Society, 2022), where pharmacists provide MTM services in these joint outpatient clinics. This marks a crucial transformation in the responsibilities of pharmacists. The results of this study indicate that patients receiving capecitabine treatment still require further medical intervention under the existing standard care model. The MTM provided by pharmacists reduces the number and severity of AEs at home for patients, highlighting the value of the oncologist-pharmacist joint service model. Multiple studies have shown that the transformation of the pharmaceutical service model has demonstrated value in various aspects such as patient compliance, patient efficacy intervention, and the incidence of AEs (Bryant et al., 2013). In a study of an oncologist-pharmacist joint outpatient clinic, the transformation of the pharmacist’s service model promoted self-management among cancer patients, thereby improving medication safety and treatment effectiveness (Liu et al., 1997). Reflecting the initial achievements of the transformation of the Chinese pharmacist’s pharmaceutical service model.

### The necessity of managing patients receiving capecitabine

In our study, the control group identified 979 cases of AEs. The intervention group, under the condition of pharmacists providing MTM services, still discovered 685 cases of AEs. This suggests that AEs are a significant challenge for patients taking oral chemotherapy drugs at home. The use of oral chemotherapy drugs is transforming the management model of cancer treatment, shifting patients from being monitored in hospitals to self-managing at home (Decker et al., 2009). Cancer patients who receive capecitabine at home are confronted with numerous medication-related risks and hazards, such as the need for self-prevention, identification, and management of AEs (Spoelstra et al., 2013). Research shows that AEs caused by oral chemotherapy drugs, such as digestive tract reactions and skin reactions, can indirectly affect the patient’s medication process by influencing their physiological and psychological conditions (Weingart et al., 2010). A study on oral chemotherapy for breast cancer found that 46% of the cases of poor medication compliance were due to AEs such as rashes, night sweats, and sleep disorders caused by the drugs (Fallowfield, 2005). The research shows that among patients with advanced colorectal cancer whose expected survival period exceeds 8 months, more than 30% of those taking oral chemotherapy drugs prematurely discontinued the treatment. The mortality rate of this group of patients was twice that of those who completed the full course of chemotherapy as prescribed by the doctor (Neugut et al., 2006). A report from the United States on medication errors involving oral chemotherapy drugs reveals that a total of 99 AEs, 322 near misses, and 87 minor harm incidents occurred at 14 cancer centers (Weingart et al., 2010).

### Measures for medication safety management of cancer patients who receive capecitabine at home

Our results indicated that 60.0% of patients received interventions during their initial appointments, highlighting the necessity for proactive screening and management of individuals undergoing capecitabine. The subsequent follow-up studies indicated that patients receiving such treatment require continuous treatment management. There have been numerous intervention studies on the management of medication safety for cancer patients receiving oral chemotherapy drugs at home. The oral chemotherapy drug monitoring clinic established by pharmacists has played a significant role in identifying medication errors, monitoring drug therapy, and managing AEs (Battis et al., 2017). Japan has developed a system for systematically managing oral chemotherapy drugs, by using promotional materials, medication treatment calendars, and treatment plan lists to assist cancer patients who are taking oral chemotherapy drugs at home, thereby promoting the safety of patients’ medication use (Komatsu et al., 2014) (Moody and Jackowski, 2010). Several medical centers have developed patient education programs to facilitate self-management among cancer patients who are receiving oral chemotherapy medication at home (Marmorat et al., 2020). It is anticipated that initiating early intervention and maintaining long-term follow-up will decrease both the frequency and severity of ADRs, ultimately enhancing patients’ quality of life and reducing healthcare expenses (Brummel et al., 2014; Rodriguez de Bittner et al., 2017). Stopping medication is also one of the safety management measures for patients. In this study, a total of 21 patients discontinued the medication due to adverse drug reactions. Among them, 6 cases occurred in the intervention group and 15 cases in the control group.

### MTM service is an effective management measure

Our research shows that the MTM services provided by the pharmacist in the joint outpatient service can significantly reduce the incidence of AEs, this is consistent with the existing research results. Herledan et al. pointed out that implementing comprehensive MTM in the oncology pharmacy clinic can effectively provide early intervention and reduce the incidence of AEs (Herledan et al., 2023). In our research, the intervention group had a significantly lower incidence of AEs, especially in anorexia and nausea. The pharmacist, by assessing the occurrence of gastrointestinal AEs in patients, suggested in the combined outpatient clinic that the doctor prescribe oral anti-nausea drugs or drugs to improve appetite for the patients, in order to alleviate their symptoms of nausea and vomiting, which is significant for optimizing treatment and patient adherence. There are also significant differences between the two groups in central and peripheral nervous system AEs, particularly in insomnia. The intervention group performs better, This is mainly related to the pharmacist’s follow-up assessment of the patient’s neurological-related AEs, and timely providing suggestions for prescribing sedative-hypnotic drugs. In hematology, the most notable AE is the decrease in hemoglobin levels. This is mainly related to the fact that pharmacists follow up on the results of patients’ hematological examinations, promptly identify patients with bone marrow suppression, and recommend that doctors take intervention measures, for example, prescribing medications to improve appetite, oral drugs to increase white blood cells, or injectable drugs for boosting white blood cell levels., in order to improve the situation of bone marrow suppression. Management of medication therapy has been employed for numerous chronic conditions. The involvement of pharmacists in MTM has enhanced clinical outcomes, reduced healthcare expenses, and augmented potential advantages (Brummel et al., 2014; Rodriguez de Bittner et al., 2017; Schultz et al., 2021; Hui et al., 2014; Nuffer et al., 2019; Zhang et al., 2021b; Shrestha et al., 2015; Germaine, 2016). Studies have shown that the MTM model has demonstrated excellent clinical, economic and humanistic benefits in the management of various chronic diseases such as asthma and cardiovascular diseases (Wang et al., 2021; Liu et al., 1997; Liu et al., 2021b). The management of MTM in patients with malignant tumors has been proven to reduce the incidence of AEs and medication errors (Herledan et al., 2023). In the joint medical clinic between oncologists and pharmacists, pharmacists can promote self-management among cancer patients through MTM services, thereby enhancing the safety of medication use and the effectiveness of treatment for these patients (DAI Yuan-yuan, 2021).

### Limitation

The research has not only highlighted the value of pharmacists in several aspects but also offered valuable insights into drug AEs and treatment optimization. However, several factors may have influenced our findings. Since the intervention group was a convenience sample of clinic patients, there might be a bias in the selection process. For instance, in terms of sampling, only patients from one hospital were included. Due to the influence of various local factors, there are significant differences in patient characteristics among hospitals, so the sample cannot represent other regions. Future research should collaborate with hospitals in different regions to obtain more generalizable data, providing support for medical policies and service improvements. Additionally, this study did not conduct a cost-benefit analysis on the two groups of patients. A study has shown, the participation of pharmacists in the emergency department leads to substantial cost savings (Aldridge et al., 2009). Cost-benefit analysis may be able to quantify the value of pharmacists in Pharmaceutical Joint Clinic. It is highly necessary to clarify this point in future studies. Finally, future research should explore intervention mechanisms and expand their clinical applications to improve treatment outcomes (Vuelta-Arce et al., 2020; Goodin et al., 2011).

## Conclusion

Therefore, in the joint outpatient service model of oncologists and pharmacists, pharmacists play a crucial role in monitoring and handling AEs. This goal is achieved through the provision of MTM services. Pharmacists can promptly assess the symptoms of patients taking capecitabine orally and quickly identify common AEs in the gastrointestinal tract, nervous system, and blood system. They can then recommend the appropriate drugs for prevention or treatment to the doctors, and provide professional drug consultation and medication guidance services to patients, helping them make correct choices and use the drugs properly, thereby reducing the incidence of AEs related to capecitabine and alleviating the severity of AEs in the gastrointestinal tract, nervous system, and blood system, thus enhancing the safety of drug use for patients.

## Data Availability

The original contributions presented in the study are included in the article/supplementary material, further inquiries can be directed to the corresponding author.
